# Focal Seizures in a Patient With Chronic Basal Ganglia Calcifications Secondary to Idiopathic Primary Hypoparathyroidism

**DOI:** 10.1210/jcemcr/luae093

**Published:** 2024-05-21

**Authors:** Jennifer Zhang, Karen Van, Patrick Carney, Christopher Gilfillan, Mathis Grossmann

**Affiliations:** Department of Endocrinology, Eastern Health, Melbourne, VIC 3128, Australia; Department of Endocrinology, Eastern Health, Melbourne, VIC 3128, Australia; Department of Neurology, Eastern Health, Melbourne, VIC 3128, Australia; Eastern Health Clinical School, Monash University, Melbourne, VIC 3128, Australia; Department of Endocrinology, Eastern Health, Melbourne, VIC 3128, Australia; Eastern Health Clinical School, Monash University, Melbourne, VIC 3128, Australia; Department of Endocrinology, Eastern Health, Melbourne, VIC 3128, Australia

**Keywords:** basal ganglia, calcifications, hypoparathyroidism, hypocalcemia, seizure

## Abstract

Patients with hypoparathyroidism can present with concurrent basal ganglia calcifications (BGCs). The exact pathogenesis is unknown, although it is thought to relate to calcium-phosphate deposition from chronic hypocalcemia and hyperphosphatemia. We present the case of a 65-year-old man with known idiopathic primary hypoparathyroidism and concurrent extensive BGC. Thirty years after diagnosis, he presented with focal seizures despite a decade of stable intracranial calcifications on imaging. Serum calcium, phosphate, 25-hydroxyvitamin D, and parathyroid hormone levels were well controlled during this period. He was commenced on lifelong levetiracetam with subsequent seizure remission. Given the scarcity of literature surrounding focal seizures and BGC, it is essential to raise awareness in this area.

## Introduction

Patients with idiopathic primary hypoparathyroidism (PHP) are generally managed with calcium and active vitamin D supplementation, aiming to maintain the serum calcium concentrations slightly below or within the low normal range, to avoid renal or other extraskeletal calcifications [1]. However, it has been postulated that hypocalcemia coupled with hyperphosphatemia may lead to a complication known as basal ganglia calcifications (BGCs) via the deposition of calcium-phosphate complexes in brain parenchymal tissue [2]. Based on limited case studies and a lack of robust prevalence data, the development of focal seizures in relation to BGC is uncommon. Moreover, there is a paucity of literature exploring seizure profiles in patients with known chronic BGC in the setting of idiopathic PHP. Although there are a myriad of case reports delineating first seizure episodes leading to incidental findings of new BGC, to the best of our knowledge, there has only been one published case report with new-onset seizures in the context of known chronic BGC, occurring in the absence of hypocalcemia [3]. In people with chronic BGC, recommendations for seizure risk stratification, the use of prophylactic antiseizure medications, and the role of brain imaging surveillance are lacking, in part, because this is an uncommonly encountered phenomenon.

## Case Presentation

We present the case of a 65-year-old male immigrant from Iran with known idiopathic PHP who presented with recurrent unconscious collapses. He had no history of neck surgery, irradiation, or significant family history of hypoparathyroidism or genetic disorders. His first witnessed and stereotyped episode occurred in July 2021, then again in March 2022. This was on a background of 3 previously unwitnessed unconscious collapses, with the first episode occurring in 2013. These stereotyped episodes would begin with intense anal pain, followed by fingertip paresthesias, unconscious collapse, then bilateral involuntary upper-limb clonic movements and frothing around the mouth before the patient regained consciousness with postictal confusion.

## Diagnostic Assessment

His initial unconscious collapse in 2013 was diagnosed as cardiogenic syncope secondary to severe hypocalcemia. This was supported by a low serum corrected calcium (1.85 mmol/L [reference range, 2.10-2.60 mmol/L]; 7.4 mg/dL) in association with a slightly prolonged QTc interval (483 ms [male reference range, 350-440 ms]). His calcitriol was increased from 0.25 mcg daily to 0.5 mcg daily, and calcium carbonate continued at 1200 mg daily. In 2016, routine biochemistry showed hypocalcemia (cCa 1.89 mmol/L; 7.58 mg/dL), and his calcium carbonate was increased to 2400 mg daily. He re-presented to another health service later in 2016 with another unconscious collapse, and mild hypocalcemia (cCa 1.99 mmol/L; 7.98 mg/dL). There was no clear etiology for this presentation and he remained on the same calcitriol and calcium carbonate doses. The patient did not have any signs of cognitive slowing or confusion and was negative for Trousseau and Chvostek signs during each of his presentations.

In 2021, during the patient's first witnessed stereotyped seizure that prompted his presentation to the emergency department, biochemistry 2 months prior to this included a low normal serum cCa (2.11 mmol/L; 8.44 mg/dL) and normal phosphate (1.01 mmol/L [reference range, 0.75-1.50 mmol/L]; 3.13 mg/dL). He self-discharged prior to consulting with a doctor and subsequently, serum studies including PTH, 25-hydroxyvitamin D, as well as computed tomography (CT) of the brain were not performed. His outpatient 24-hour Holter monitoring and electroencephalogram (EEG) did not yield any abnormal findings. The patient's subsequent witnessed episode in 2022 also yielded unremarkable results in his cCa (2.26 mmol/L; 8.44 mg/dL) and phosphate (1.7 mmol/L; 5.26 mg/dL).

**Figure 1.**
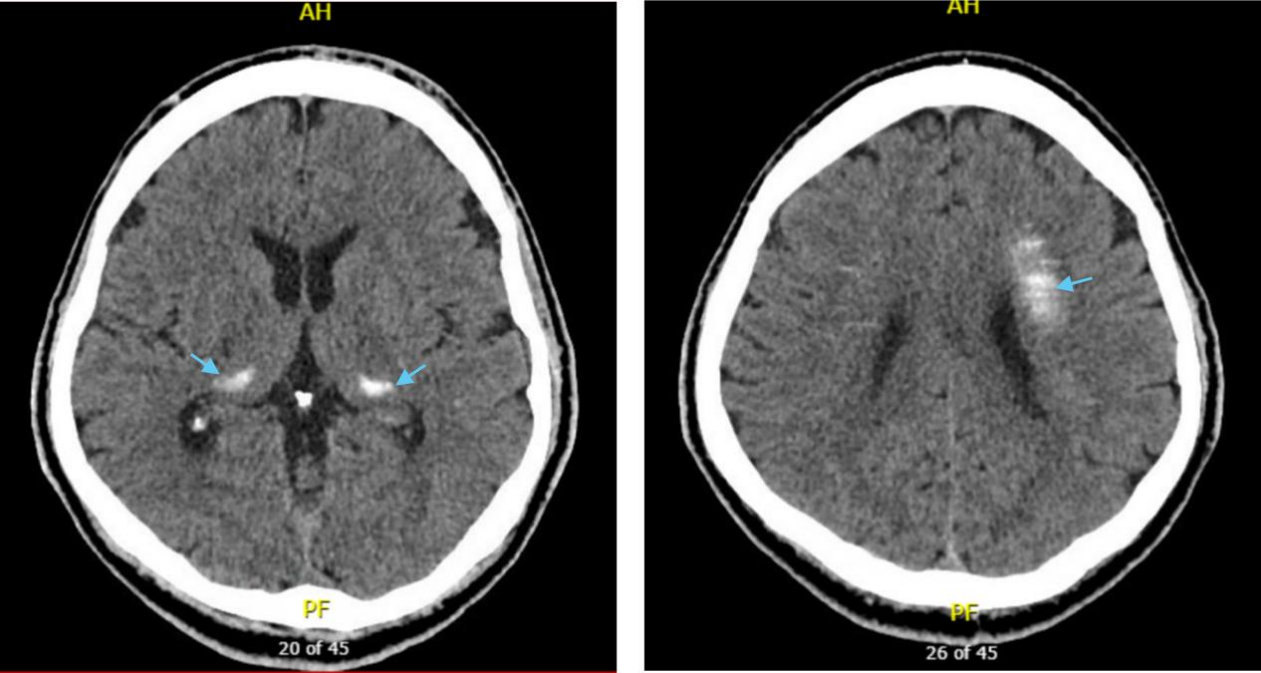
Noncontrast axial computed tomography images of the head in 2013 showed calcifications in the bilateral basal ganglia, including the globus pallidus, posterior aspect of thalami, dentate nuclei, and corona radiata.

**Figure 2.**
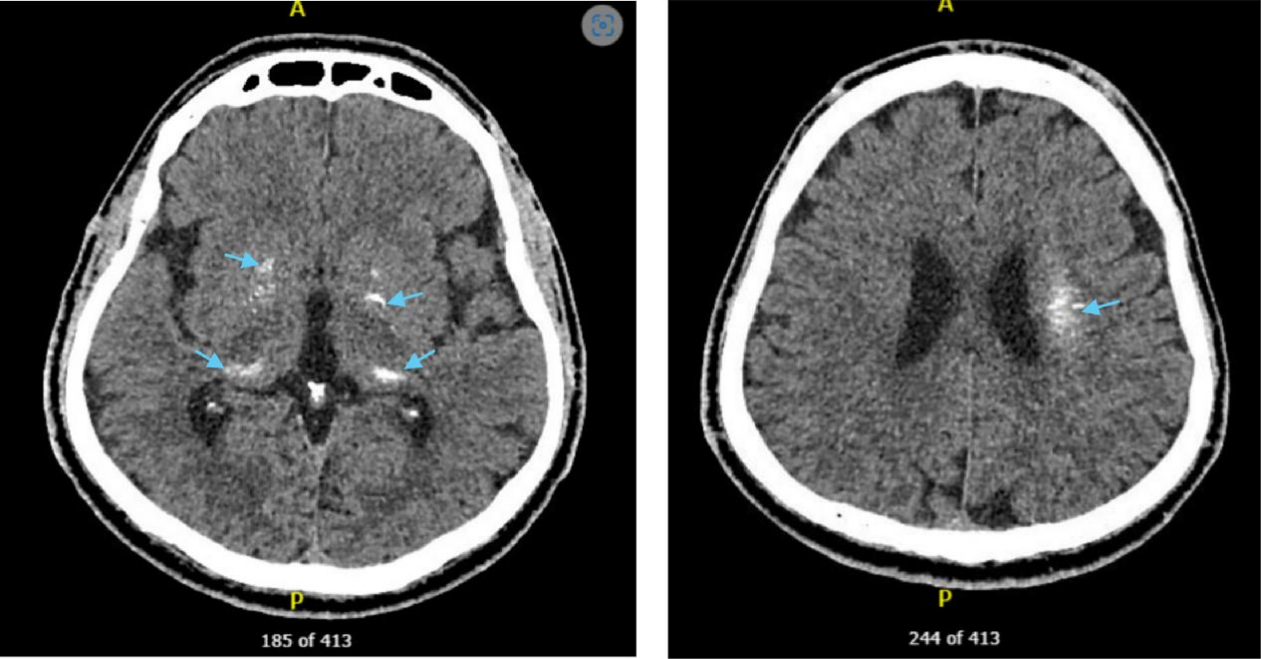
Noncontrast axial computed tomography images of the head in 2022 showed calcifications in the bilateral basal ganglia, left centrum semiovale, and dentate nuclei of the cerebellum.

His previous CT brain from 2013 **(**Fig. 1) was reviewed, and interestingly, already showed extensive calcifications bilaterally in the globus pallidus, posterior aspect of thalami, dentate nuclei, and corona radiata. Repeat CT of the brain in 2022 (Fig. 2) showed no progression of intracranial calcifications. Magnetic resonance imaging (MRI) of the brain in 2023 demonstrated extensive symmetric calcification of the basal ganglia, dorsal thalami, dentate nuclei, and deep medullary veins of the supratentorial white matter. However, this was unable to be reliably compared to 2017 due to the use of T1-weighted images only. The neurologist's opinion was that despite the atypical features of these episodes, they most likely reflected unprovoked epileptic seizures given the patient’s normocalcemia. The BGCs were also thought unlikely causative for his seizures, and he was presumed to have a lesion-negative focal epilepsy. Furthermore, a normal EEG is common in patients with epilepsy and is not required for the diagnosis. A single routine EEG also has less than 50% sensitivity for detection of interictal epileptiform discharges, especially if performed greater than 24 hours after a seizure [4].

## Treatment

The patient had remained on calcitriol 0.5 mcg daily and calcium carbonate 1200 mg daily from 2013 until 2016, from which time his calcium carbonate was increased to 2400 mg daily due to intermittent severe hypocalcemia. Since 2017, the patient's calcitriol and calcium carbonate were adjusted with the aim of mild hypocalcemia to normocalcemia. These targets have been achieved with minimal dose adjustments. The patient was also commenced on levetiracetam in 2022, with no further stereotyped episodes since then.

## Outcome and Follow-up

The patient had clinic follow-up appointments every 6 months with his endocrinologist and neurologist. His latest serum cCa (2.09 mmol/L; 8.38 mg/dL) and phosphate levels (1.16 mmol/L; 3.59 mg/dL) remained low normal and high normal, respectively. His 24-hour urine calcium level was 6.8 mmol/day (reference range, 0.1-7.5 mmol/day), PTH, and vitamin D levels were 0.9 pmol/L (reference range, 1.6-1.9 pmol/L) and 73 nmol/L (reference range >50 nmol/L), respectively. Renal tract ultrasound in 2016 and 2022 did not show nephrocalcinosis nor nephrolithiasis, and estimate glomerular filtration rate was 58 mL/min/1.73m^2^ (reference range >90 mL/min/1.73m^2^). The patient has remained clinically stable, asymptomatic, and seizure free since March 2022.

## Discussion

BGCs are a common complication of idiopathic PHP, with a prevalence of 51% to 74% [5, 6]. This is contrasted against the prevalence of BGCs in the general population being suggested top range between 2% and 12.5% [7], depending on the study. Of note, BGCs may be found more commonly in the older population, with 43.5% of patients presenting to a memory clinic found to have BGC [8]. Within this group, half were deemed to have “mild” BGC. Additionally, physiological BGC is usually asymptomatic. The most commonly affected sites include the globus pallidus, putamen, and caudate nuclei (68.8%, 55.9%, and 54.8%, respectively) [2]. The pathogenesis of BGC in the setting of hypoparathyroidism is unclear. The hypothesis is that persistent hypocalcemia, hyperphosphatemia, and poor calcium control promote calcium-phosphorus complex deposition in brain parenchymal tissue [2, 9]. Low PTH levels in idiopathic PHP may also have a role. PTH-dependent sodium-phosphate transporters are expressed in the basal ganglia, and loss of PTH effect on these may facilitate intracellular phosphate deposition, forming a nidus for calcium hydroxyapatite deposition [10].

Our literature search demonstrated no clear association between the degree of BGC and the prevalence of seizure presentations. A case-controlled study of 166 patients with incidental findings of BGC compared with 622 controls suggested that BGC is unlikely to cause seizures due to the odds ratio of epilepsy being only 0.9 in the former group [11]. This notion is reinforced in other case reports, with the lack of association observed between seizures and cerebral calcifications in patients with chronic hypoparathyroidism [12]. These findings support the unexpected nature of new focal seizures developing in our case, wherein the intracranial calcifications had remained stable for a decade. Interestingly, in a large case series of 145 patients with idiopathic PHP, a history of seizures was present in 60.7% and was a significant predictor for BGC and its progression [2]. However, selection bias exists within this high incidence, due to the mean duration of hypocalcemia and/or hyperphosphatemia occurring for up to 15 years in many of these cases [2]. The presence of longstanding electrolyte imbalances are a confounding factor for seizure occurrence in the setting of concurrent BGC. Thus, the association between BGC and seizures is largely inconclusive.

There is even less literature exploring seizure profiles in patients with hypoparathyroidism and known chronic BGC. To the best of our knowledge, the only case report to have explored this included a patient who developed new focal seizures in the setting of a 30-year history of BGC [3]. There was progression of calcification from caudate and lentiform nucleus to the corona radiata, thalamus, subcortical frontal, and parietal lobes when comparing CT of the brain and skull x-ray from 1990 to previous skull x-rays in 1981 and 1986. Unfortunately, there was no prior CT brain imaging to adequately ascertain interval changes using the same modality. Serum calcium and phosphate levels were adequate throughout these periods, subsequently removing electrolyte imbalance as an implicating factor. This case remains different from ours, in that we can state with near certainty that there was no progression of intracranial calcification. Given the scarcity of literature surrounding this subset of patients, it is no surprise that there have been few advances in associating the likelihood of seizure activity in those with known chronic BGCs.

Our report highlights the lack of certainty in predicting those with BGC who will progress to develop seizures in idiopathic PHP. There are currently no published clinical guidelines or consensus statements for this subset of patients, especially when seizure risk is largely unknown even when compared with that of the general population. The use of PTH analogues to combat hypocalcemia in PHP has been previously been explored but long-term efficacy data are lacking. Thus, it is usually reserved as an adjunct for patients whose calcium levels are not well controlled by conventional medical therapy. Future large prospective studies exploring seizure activity in association with the location and extent of BGCs are recommended. Additionally, the frequency of imaging surveillance, and the role of prophylactic antiseizure medications, should concurrently be explored to enable a well-rounded approach to the management of these patients.

## Learning Points

- An initial CT or MRI brain should be considered in all patients with hypoparathyroidism to assess for baseline intracranial calcifications. This will provide a referral point in the situation where patients progress to developing seizures. It should be noted that the yield of this may not be as high as we hope due to the current unclear association between BGC and seizure activity. The temporal progression of intracranial calcifications in relation to seizures would require exploration in large prospective trials to ascertain potential correlations.
- There is currently no definitive clinical evidence that the presence or progression of BGC increases the risk of seizures.
- In patients with known idiopathic PHP who present with seizure and/or unconscious collapses, differentials of severe hypocalcemia and BGC/intracranial calcifications should be considered.
- There are currently no guidelines for radiological interval surveillance or pharmacological management in patients with seizures secondary to known BGC; hence, clinical judgment and individualization of management is essential. Future studies will hopefully address these gaps in management.

## Data Availability

Original data generated and analyzed during this study are included in this published article.

## Abbreviations

BGC: basal ganglia calcification
cCa: corrected calcium
CT: computed tomography
EEG: electroencephalogram
MRI: magnetic resonance imaging
PHP: primary hypoparathyroidism
